# Neonatal diabetes mellitus is a significant feature of COXPD‐24 caused by recessive *NARS2* variants

**DOI:** 10.1111/dme.70129

**Published:** 2025-08-31

**Authors:** Russell Donis, Matthew N. Wakeling, Nicola Jeffery, Molly Govier, Matthew B. Johnson, Samar Sabir Hassan, Mohammed Ahmed Abdullah, Khadiga Yehia Elsayed Eltonbary, Nima Parvaneh, Mahsa M. Amoli, Farzaneh Abbasi, Selin Elmaoğulları, Semra Çetinkaya, Kubra Gunes, Meltem Tayfun, Hanieh Yaghootkar, Andrew T. Hattersley, Sarah E. Flanagan, Elisa De Franco

**Affiliations:** ^1^ Department of Clinical and Biomedical Science University of Exeter Medical School Exeter UK; ^2^ Department of Pediatric Endocrinology Gaafar Ibn Auf Pediatric Tertiary Hospital Khartoum Sudan; ^3^ Sudan Childhood Diabetes Center Khartoum Sudan; ^4^ Department of Paediatrics and Child Health, Faculty of Medicine University of Khartoum Khartoum Sudan; ^5^ Department of Pediatrics, Faculty of Medicine Ain Shams University Cairo Egypt; ^6^ Department of Pediatrics Dr. Soliman Fakeeh Hospital Saudi Arabia; ^7^ Division of Allergy and Clinical Immunology, Pediatrics Center of Excellence, Children's Medical Center Hospital Tehran University of Medical Sciences Tehran Iran; ^8^ Metabolic Disorders Research Center, Endocrinology and Metabolism Molecular‐Cellular Sciences Institute Tehran University of Medical Sciences Tehran Iran; ^9^ Dr. Sami Ulus Children's Training and Research Hospital, Clinic of Pediatric Endocrinology Ankara Turkey; ^10^ University of Health Sciences, Turkey Ankara Turkey; ^11^ Department of Pediatrics Tokat State Hospital Tokat Turkey; ^12^ Sincan Eğitim Araştırma Hastanesi Pediatric Endocrinology Department Ankara Turkey; ^13^ Joseph Banks Laboratories, College of Health and Science University of Lincoln Lincoln UK

**Keywords:** mitochondria, mitochondrial disease, mitochondrial dysfunction, monogenic diabetes, NARS2, neonatal diabetes mellitus, β‐Cells

## Abstract

**Background:**

Recessive loss‐of‐function *NARS2* variants causing the multi‐system disorder Combined oxidative phosphorylation deficiency 24 (COXPD24) have recently been reported in 3 individuals with diabetes diagnosed between 3 days and 14 months of age. In this study, we investigate the presence of *NARS2* variants in a large cohort of individuals with early‐onset diabetes.

**Methods:**

We used genome and targeted next‐generation sequencing to screen for rare, coding biallelic *NARS2* variants in a cohort of 397 individuals diagnosed with diabetes <24 months of age of unknown genetic cause.

**Results:**

We identified 8 individuals with homozygous disease‐causing missense variants in *NARS2* (4 individuals with the p.(Phe216Leu) variant, 3 with p.(Thr180Asn) and one with p.(Val440Leu)).

All 8 individuals were diagnosed with insulin‐dependent diabetes before 6 months of age (neonatal diabetes, NDM) with the median age at diagnosis being 4 weeks (range: 1 to 20 weeks). 7/8 probands had low birthweight (median *Z*‐score: −2.43, range: −4.17 to 0.86). Neurological features were common, with epilepsy and developmental delay each identified in 7/8 and 6/8 participants, respectively.

**Conclusion:**

Taken together with previously published cases, this study shows that NDM is an important feature of COXPD‐24 and highlights a critical role for NARS2 in the insulin‐secreting pancreatic β‐cell.

**What is new?**

- Mitochondrial function is important for the β‐cell. Recently, variants in the gene encoding the mitochondrial protein NARS2 have been linked to neonatal/early‐onset diabetes in 2 families.
- In our cohort of 397 children with diabetes onset <2 years without a known genetic cause, we identified 8 individuals with homozygous *NARS2* variants. All were diagnosed in the first 6 months of life and had additional neurological features including epilepsy and developmental delay.
- Our results confirm *NARS2* variants as a cause of syndromic neonatal diabetes, which should be considered when performing genetic testing for this condition.

## 1 INTRODUCTION

Mitochondria are essential for the function of pancreatic β‐cells as they provide the ATP required to facilitate insulin secretion and synthesise key metabolites coupling glucose sensing to insulin granule exocytosis.^1^ Consistent with this, genetic variants affecting mitochondrial function, for example the mitochondrial DNA m.3243A>G variant, can cause monogenic diabetes.^2^

Recently, pathogenic variants in the nuclear gene, *NARS2*, which encodes an aminoacyl‐tRNA synthetase critical for protein synthesis in the mitochondria, have been identified in 3 individuals (including 2 siblings) with diabetes diagnosed between 3 days and 14 months of age.^3^, ^4^ These reports were the first descriptions of diabetes in individuals with biallelic variants in *NARS2*, which are known to cause Combined oxidative phosphorylation deficiency 24 (COXPD‐24; OMIM: 616239). This multiorgan condition predominantly affects the muscles and central nervous system (CNS) and has been reported in 37 individuals in the literature to date.^5^

Neonatal diabetes (NDM) is a monogenic form of diabetes usually diagnosed before 6 months of age, with some rare cases manifesting later. There are currently >40 known genetic causes.^6^ Extra‐pancreatic features are often detected in individuals with NDM, with approximately 18% of cases having neurological features such as developmental delay, epilepsy, or structural brain malformations.^7^ A genetic diagnosis of neonatal diabetes is critically important as it guides medical management and treatment,^8^, ^9^ as well as informing genetic counselling in the participants' families.

The aim of this study was to identify recessive *NARS2* disease‐causing variants in individuals with genetically unsolved diabetes diagnosed in the first 2 years of life.

## 2 METHODS

### 2.1 Study design and cohort

Informed consent was obtained from the parents/guardians for genetic testing of each patient, with the study approved by the North Wales Research Ethics Committee (517/WA/0327). Clinical and demographic information was collected through a standardised referral form and from the participants' medical records.

From a cohort of 3763 individuals with diabetes diagnosed before 2 years of age (2890 diagnosed before 6 months) referred to the Exeter Genomics Laboratory for genetic testing between January 2000 and July 2024, we selected individuals who met 2 criteria: (1) no disease‐causing variant had been identified following comprehensive testing of the known genetic causes of neonatal and early‐onset diabetes (see *Genetic analysis of known NDM genes* section) and (2) targeted next‐generation sequencing or genome sequencing data covering the *NARS2* gene was available for analysis. 397 individuals (249 females, 148 males) referred from 71 countries met both criteria and were therefore included in the study. 256/397 were diagnosed before the age of 6 months and were classified as having NDM. Diabetes remission had been reported in 31 individuals with NDM, who were therefore diagnosed with transient NDM; whilst the other 225 had permanent NDM. Ninety‐five individuals (62 with NDM) were born to related parents. Sixty‐three (39 with NDM) had neurological features. Twenty‐six individuals were born to consanguineous parents and had neurological features, including 14 with NDM.

### 2.2 Genetic analysis of known genetic causes of neonatal and early‐onset diabetes

All known genetic causes of NDM had been excluded in the 397 individuals using targeted next‐generation sequencing (tNGS) of the known etiological genes^10^ (Table S1). Methylation‐specific MLPA analysis of the chr6q24 transient neonatal diabetes locus had been performed in all individuals diagnosed with diabetes before 6 months of age (SALSA MLPA Probemix ME033 TNDM, MRC Holland, Amsterdam, The Netherlands).

### 2.3 Genetic analysis of *NARS2*

Sequence analysis of the *NARS2* gene was performed using genome sequencing (BGI DNBSeq technology, *n* = 277) or tNGS (*n* = 120). The list of genes included in the tNGS panel is provided in Table S1. For the tNGS, RNA baits targeting the 14 exons and intron‐exon boundaries of *NARS2* (NM_024678) were added to a custom‐designed in‐solution capture assay.^10^ Sequencing was performed on an Illumina NextSeq 500 platform.

Bioinformatic analysis of tNGS and genome sequencing data was performed as previously described^11^ using a range of software packages. Sequence reads were aligned to the GRCh37/hg19 human reference genome using the Burrow‐Wheeler Aligner for short reads (BWA‐MEM v0.7.15) followed by local re‐alignment using the Broad Institute's GATK IndelRealigner (v3.7.0). Variants were called using the GATK haplotype caller and annotated using Alamut Batch (Interactive Biosoftware v1.11, Rouen, France). Copy number variants were called by SavvyCNV,^12^ a software developed in‐house which uses read depth to detect heterozygous and homozygous copy number variations.

Homozygous and compound heterozygous *NARS2* coding variants and variants affecting the canonical splice sites with an allele frequency below 0.01% in gnomAD_v4^13^ were prioritised for analysis and classified according to the American College of Medical Genetics and Genomics (ACMG) guidelines.^14^ Missense variants were assessed in silico using the bioinformatic tool REVEL.^15^ Testing of family members for the *NARS2* variants was performed using tNGS or genome sequencing data. Parental samples were not available for testing from Family 5, 6 or 8, and only maternal samples were available from Family 4 and 7.

### 2.4 Homozygosity analysis

In one proband with a homozygous *NARS2* variant but without self‐reported parental consanguinity, genome sequencing data was interrogated to assess the likelihood of two variants being identical by descent. Calculation of total genomic homozygosity (regions ≥3Mbp) was performed as previously described,^16^ with consanguinity defined as having genomic homozygosity ≥3% (which is the cut‐off recommended by the ACMG based on average percentage of homozygosity for the offspring of second cousins^17^).

## 3 RESULTS

### 3.1 Molecular genetics

**FIGURE 1:**
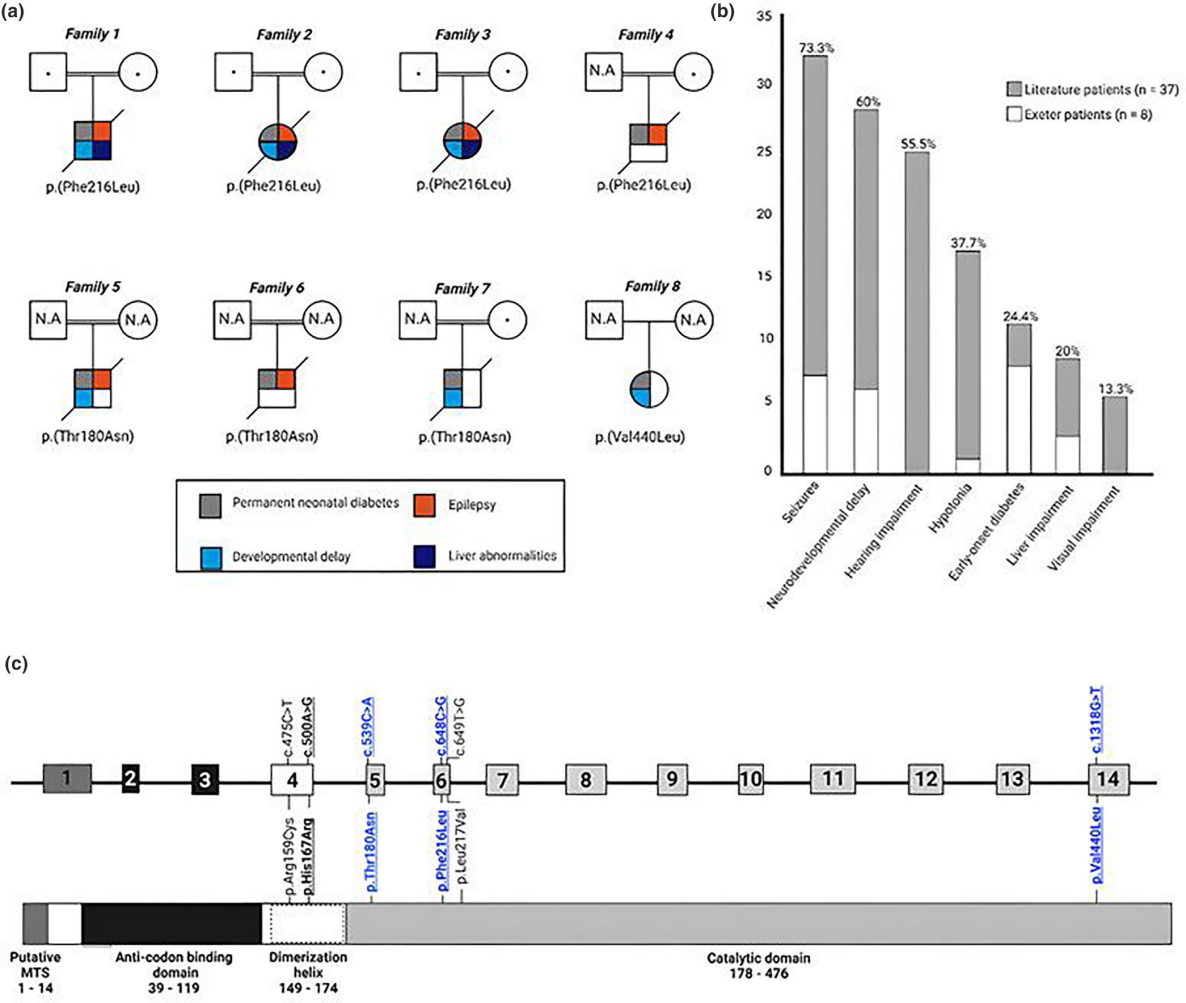
Identification of 8 participants with neonatal diabetes caused by *NARS2* recessive variants confirms diabetes as a significant feature of COXPD‐24. (a) Partial pedigrees of the 8 participants with neonatal diabetes and additional clinical features caused by homozygous *NARS2* variants identified in this study. Symbols with dots indicate carriers. (b) Summary of clinical features of the 45 individuals with biallelic *NARS2* variants reported to date (37 cases reported in the literature and the 8 cases reported here). Only features present in ≥5 individuals are shown. Percentages at the top of bars represent the proportion of the cohort (*n* = 45) with that feature. (c) Schematic representation of the *NARS2* gene (top) and protein (bottom) labelled with biallelic *NARS2* variants identified in 11 individuals with diabetes. Bold and underlined variants are homozygous. Variants in blue are the variants identified in this study. MTS = mitochondrial targeting signal; N.A = sample not available for testing.

We identified homozygous *NARS2* missense variants (Figure 1a) in 8/397 (2%) individuals with diabetes diagnosed <24 months of age. Three different variants were identified: p.(Phe216Leu) in 4 individuals, p.(Thr180Asn) in 3 individuals and p.(Val440Leu) in one proband. No other rare, homozygous or compound heterozygous *NARS2* variants were detected in the remaining 389 individuals.

Both parents were confirmed as heterozygous carriers of the p.(Phe216Leu) variant for participants 1, 2 and 3. The mothers of patient 4 and patient 7 were heterozygous carriers for the p.(Phe216Leu) and p.(Thr180Asn) variants, respectively. Samples for the fathers of patient 4 and patient 7 were not available for testing. The samples for participants 5, 6 and 8's parents were not available for testing (Figure 1a).

Parental consanguinity was reported at referral for 7/8 probands. Total genomic homozygosity for the 8th individual was 1.87%, thereby excluding close parental relatedness. The *NARS2* variant was, however, found to be located in the largest homozygous region identified in this individual (22.37 Mb). There are two possible explanations for this result: either the biallelic variant is in a region identical by descent due to the parents being distantly related, or the 22.37 Mb homozygous region resulted from segmental, uniparental isodisomy of this region on chromosome 11. Parental samples from this patient were not available for testing, and we are therefore unable to confirm which one of these events underlies the presence of the homozygous *NARS2* variant in this individual. Three individuals homozygous for the p.(Phe216Leu) variant and two with the p.(Thr180Asn) variant were referred from the same country (Sudan and Iran, respectively). Whilst these families were not reported to be related at referral, it is possible that some of the patients may share a common ancestor.

Using all available evidence and following the ACMG variant classification guidelines,^14^ we classified the 3 missense variants identified in this study as likely pathogenic (details on variant classification are summarised in Table S2). This result is consistent with a diagnosis of Combined oxidative phosphorylation deficiency 24 (COXPD24; OMIM: 616239) due to recessively inherited *NARS2* variants in our 8 participants.

### 3.2 Clinical characteristics of individuals with *NARS2* biallelic variants

Seven of eight individuals with a *NARS2* disease‐causing variant in our study had low birthweight with a median *Z*‐score of −2.43 (*Z*‐score range −4.17 to −0.86), consistent with reduced insulin secretion in utero.^18^ All 8 individuals had NDM (median age at diagnosis of diabetes = 4 weeks; range: 1 to 20 weeks) with median blood glucose levels at presentation of 33.25 mmol/L (range 11.1 to 46.9 mmol/L, data available for 7/8 individuals). Serum C‐peptide was measured in 3/8 individuals and was low in each case (range 0.009 to 0.076 nmol/L). All 8 probands were treated with insulin since diagnosis (median dose = 1.1 units/kg/day, range 0.3 to 2), with no diabetes remission periods reported.

All individuals had neurological features which included epilepsy (88%, *n* = 7/8) and/or developmental delay (75%, *n* = 6/8). Other extra‐pancreatic features such as liver abnormalities (38%, *n* = 3/8) and/or renal abnormalities (25%, *n* = 2/8) were also reported. Seven of the eight individuals died before the age of 15 months, with sepsis being the most common cause of death (57%, *n* = 4/7). The 8th individual was lost to follow up after referral for genetic testing at 18 days. None of the participants in this study received mitochondrial supportive therapy. A summary of the clinical features of the 8 individuals is provided in Figure 1b and Table 1.

**TABLE 1:** Clinical characteristics of 11 individuals with diabetes and biallelic variants in *NARS2*.

Patient	*NARS2* variants	Sex	Country	Birthweight /gestation (*Z*‐score)	Age at diagnosis (wks)	Glucose presentation (mmol/L)	Age at last assessment	C‐peptide (nmol/L)	Insulin dose (U/kg/day)	Neurodevelopmental features	Parents related	Other features
P1 (this study and Hassan et al^19^)	c.648C>G, p.(Phe216Leu) (homozygous)	M	Sudan	2500 g/38 wks (−1.51)	2	37	3 months (deceased)	N/A	1	DD, epilepsy	Yes	Anaemia, coarse features with protruding tongue, abnormal LFTs, thyroid dysfunction, umbilical hernia. Cause of death: sepsis
P2 (this study)	c.648C>G, p.(Phe216Leu)‐ (homozygous)	F	Saudi Arabia	1500 g/38 wks (−4.17)	2	36.3	4 months (deceased)	0.0563	0.7	DD, epilepsy	Yes	Abnormal LFTs. Cause of death: cardiac arrest.
P3 (this study and Hassan et al^19^)	c.648C>G, p.(Phe216Leu) (homozygous)	F	Sudan	2300 g/NR	20	28	4 months (deceased)	N/A	On insulin, dose N/A	DD, epilepsy	Yes	Hepatomegaly, fluctuating hypo and hyperglycaemia. Cause of death: sepsis
P4 (this study and Hassan et al^19^)	c.648C>G, p.(Phe216Leu) (homozygous)	M	Sudan	2000 g/38 wks (−2.75)	4	22.2	2 months (deceased)	N/A	0.5	Epilepsy	Yes	Chronic diarrhoea, abdominal distension, recurrent hypos with hyperglycaemia, rickets. Cause of death: sepsis
P5 (this study)	c.539C>A, p.(Thr180Asn) (homozygous)	M	Iran	2350 g/NR	6	55	4 months (deceased at 7 months)	N/A	0.38	DD, epilepsy, progressive neurological impairment	Yes	Muscle weakness. Cause of death: progressive neurological impairment and loss of consciousness.
P6 (this study)	c.539C>A, p.(Thr180Asn) (homozygous)	M	Iran	1900 g/34 wks (−0.86)	8	N/A, presented in DKA	6 months (deceased)	N/A	0.3	Epilepsy	Yes	Hydronephrosis of left kidney. Cause of death: multiple organ damage due to disseminated intravascular coagulation.
P7 (this study)	c.539C>A, p.(Thr180Asn) (homozygous)	M	Turkey	2210 g/39 wks (−2.72)	3	46.9	4 months (deceased)	0.0166	1	DD, epilepsy, cortical dysplasia	Yes	Anaemia, tubulopathy. Cause of death: sepsis.
P8 (this study)	c.1318G>T, p.(Val440Leu) (homozygous)	F	Turkey	1810 g/37 wks (−2.57)	1.57	11.1	18 days (lost to follow up)	0.0099	1.8	DD	No	NR
Yagasaki et al.^4^	c.475C>T, p.(Arg159Cys)/c.649 T>G, p.(Leu217Val)	F	Japan	2192 g/39 wks (−2.50)	12	31.6	3 years	0.076	CSII (sulfonylurea not effective)	Epilepsy	No	Severe metabolic acidosis, lactic acidosis, hearing loss, severe spastic quadriplegia, tachycardia, Kussmaul breathing, cortical atrophy
Yagasaki et al.^4^	c.475C>T, p.(Arg159Cys)/c.649 T>G, p.(Leu217Val)	M	Japan	1868 g/37 wks (−2.56)	0.43	19	1 year	0.033	CSII (withdrawn from insulin at 6 months old)	DD, epilepsy	No	Hearing loss, metabolic acidosis, lactic acidosis, brain atrophy
Cokayaman et al.^3^	c.500A>G, p.(His167Arg) (homozygous)	F	Turkey	2650 g/36 wks (0.11)	56	89.8	14 months	NR	NR	DD, epilepsy, large subdural haemorrhage	Yes	Hearing loss, hypotonia, lactic acidosis

Abbreviations: CSII, continuous subcutaneous insulin infusion; DD, developmental delay; DKA, diabetic ketoacidosis; F, female; LFTs, liver function tests; M, male; N/A, not available; NR, not recorded; wks, weeks. *Note*: Variants are annotated against the *NARS2* MANE select transcript (NM_024678, ENST00000281038, RefSeq NR_189156).

### 3.3 Frequency of diabetes in individuals with COXPD‐24

Eight individuals with NDM caused by homozygous *NARS2* variants (Figure 1c) in our cohort had features consistent with COXPD‐24. Three cases with *NARS2* recessive variants and diabetes have been reported in the literature, including two siblings with compound heterozygous variants^4^ and an unrelated individual with a homozygous missense variant who was diagnosed with diabetes at 14 months.^3^ Diabetes has therefore now been identified in a total of 11 cases from 45 reported in the literature, thereby affecting 24% of known cases with disease‐causing *NARS2* variants.

## 4 DISCUSSION

We report 8 individuals with NDM caused by homozygous *NARS2* variants. This is the largest cohort of individuals with diabetes caused by *NARS2* variants to be added to the literature, highlighting early‐onset diabetes as an important feature of COXPD‐24.

Our findings establish diabetes as a significant clinical feature of COXPD‐24, present in at least 24% (11/45) of cases reported to date. All 11 individuals with diabetes caused by *NARS2* variants (our 8 cases, 2 siblings^4^ and one individual diagnosed with diabetes at 14 months^3^) were insulin‐dependent and most had low birthweight, consistent with insulin deficiency in utero and post‐birth.^18^ Seven of our participants were treated with insulin until their death and the 8th patient was lost to follow‐up, thus it is not known if the diabetes had persisted. Unlike our participants, the previously reported siblings with *NARS2* variants and NDM were treated with mitochondrial supportive therapy and were alive at 1 and 3 years of age.^4^ Insulin treatment was stopped in the former individual at 6 months of age after normalisation of blood glucose levels.

In addition to the aforementioned individuals, one report of a 2‐month‐old infant with compound heterozygous variants in *NARS2* (a heterozygous deletion of exons 8–9 and the p.(Pro214Leu) missense variant^20^) mentioned hyperglycaemia in the neonatal period and other features indicative of a mitochondrial disorder, including lactic acidosis. Although no information on the duration of hyperglycaemia or treatment was available for this case, this observation further supports diabetes being a significant feature of COXPD‐24.

The 8 individuals with homozygous disease‐causing *NARS2* variants identified in this study had neurological features and other extra‐pancreatic features in addition to diabetes. Epilepsy and neurodevelopmental delay are the most common neurological features to be reported in COXPD‐24, affecting 73% (27/37) and 56.8% (21/37) of individuals, respectively.^3^, ^4^, ^5^, ^20^, ^21^, ^22^, ^23^, ^24^, ^25^, ^26^, ^27^, ^28^, ^29^, ^30^, ^31^, ^32^, ^33^, ^34^ Epilepsy and neurodevelopmental delay were also common in our cohort, observed in 88% (7/8) and 75% (6/8) of cases, respectively. Other extra‐pancreatic features identified in our cohort included liver and renal abnormalities, observed in 37.5% (3/8) and 25% (2/8) of individuals, respectively. This is consistent with the phenotype previously observed in other individuals with COXPD‐24 (OMIM: 616239) who had a complex, multi‐system disorder with variable phenotypes and severity.

In our discovery cohort of 397 individuals with early‐onset diabetes of unknown genetic cause, 39 had NDM and neurological features. Of these, 8 individuals had a homozygous disease‐causing *NARS2* variant, suggesting that *NARS2* variants are a significant cause of NDM with neurological features in individuals without a genetic diagnosis (20.5% of cases). Further, when taking consanguinity into account, 50% (7/14 individuals with NDM and neurological features who were born to consanguineous parents) had a homozygous disease‐causing *NARS2* variant, highlighting the need to test for this gene in individuals with NDM, especially if born to related parents and when neurological features are present.

*NARS2* encodes an aminoacyl‐tRNA synthetase (aRS2) which catalyses the ligation of the amino acid asparagine to its cognate tRNA during protein translation in the mitochondria.^35^ Consistent with its key cellular function, *NARS2* mRNA is widely expressed in human fetal and adult tissues (including pancreas) (GTEx^36^), as is the mouse orthologue *nars2* in mouse embryonic and adult tissues (GXD database, https://www.informatics.jax.org/mgihome/GXD). In vitro studies in fibroblasts from individuals with COXPD‐24 who did not have diabetes showed decreased amounts of charged mt‐tRNA^Asn^, decreased oxygen consumption, and decreased electron transport chain (ETC) activity.^20^, ^23^, ^31^ Other studies performed on muscle biopsies^21^, ^24^, ^27^ also observed a decrease in ETC activity. Consistent with this, homozygous *Nars2* knockout in mice results in failure to gastrulate and complete embryonic lethality before organogenesis.^37^ These findings are consistent with the essential role of NARS2 in mitochondrial protein translation and cell function.

The identification of diabetes as a common feature in individuals with COXPD‐24 suggests an important role for NARS2 in pancreatic β‐cells. NDM has now been reported in individuals with disease‐causing variants in 3/16 aRS2 genes^35^ listed in OMIM.^38^ Besides *NARS2*, the 2 other genes are *SARS2* (diabetes reported in 2/8 individuals^39^, ^40^) and *TARS2* (5/32 individuals^41^, ^42^). The aRS2 genes encode mitochondrial tRNA synthetases, which are essential for protein translation in the mitochondria. The m.3243A>G variant in the *MT‐TL1* gene, encoding the mitochondrial tRNA mt‐tRNA^Leu^, causes maternally inherited diabetes and deafness (MIDD), a form of monogenic diabetes characterised by diabetes onset between 11 and 68 years of age.^43^ The m.3243A>G variant impairs mitochondrial protein synthesis, leading to defective translation of the mtDNA‐encoded respiratory chain complex proteins and ultimately resulting in impaired oxidative phosphorylation.^44^ Further studies will be needed to understand how disruption of mitochondrial protein translation by the m.3243A>G variant and variants in *NARS2*, *SARS2*, and *TARS2* affects the β‐cell, leading to monogenic diabetes.

Biallelic *NARS2* variants have been reported to cause a wide spectrum of clinical presentations, ranging in severity from early‐onset COXPD‐24 to non‐syndromic deafness.^28^ Additional studies of large cohorts will be needed to understand whether there is any genotype–phenotype correlation and which factors may influence the phenotypic variability.

Our study confirms *NARS2* as an NDM etiological gene, which should be tested in neonates presenting with hyperglycaemia, especially if they are born to consanguineous unions and have neurological features. The identification of 8 probands with NDM caused by biallelic *NARS2* variants in our study brings the total number of individuals with COXPD‐24 and diabetes to 11 (24%), establishing neonatal and early‐onset diabetes as a clinical feature of this disorder.

## AUTHOR CONTRIBUTIONS

E.D.F. conceived and designed the study. A.T., I. A., J.A., J.Y., M.A., H.Y., A.T.H., S.E.F., E.D.F., and M.Y. were responsible for referrals for genetic testing and for providing and collecting clinical data. M.W. performed initial processing of WGS data. R.D., M.B.J., and E.D.F. analysed and interpreted WGS and tNGS data. R.D. curated the clinical information and contacted clinicians for follow‐up, created display items and wrote the first manuscript draft, which was critically revised by E.D.F. and S.E.F. with input from all authors.

## FUNDING INFORMATION

RD is the recipient of a Diabetes UK PhD Studentship (20/0006237). ATH is employed as a core member of staff within the National Institute for Health Research–funded Exeter Clinical Research Facility and is an NIHR Emeritus Senior Investigator. SEF has a Wellcome Trust Senior Research Fellowship (223187/Z/21/Z). EDF is a Diabetes UK RD Lawrence Fellow (19/005971) and the recipient of a Future Leader Award funded by the European Association for the Study of Diabetes and Novo Nordisk. MBJ is funded by a Diabetes UK/Breakthrough T1D RD Lawrence Fellowship (23/0006516). This study was supported by the National Institute for Health and Care Research Exeter Biomedical Research Centre. The views expressed are those of the author(s) and not necessarily those of the NIHR or the Department of Health and Social Care.

The funders had no role in study design, data collection and analysis, decision to publish, or preparation of the manuscript. This research was funded in whole, or in part, by Wellcome (223187/Z/21/Z and 108101/Z/15/Z). For the purpose of open access, the author has applied a CC BY public copyright licence to any Author accepted Manuscript version arising from this submission.

## CONFLICT OF INTEREST STATEMENT

No potential conflict of interest relevant to this article was reported.

## Supporting information


**Table S1.** Genes included in the targeted next‐generation sequencing panel for neonatal diabetes mellitus.
**Table S2.** Variant classification of recessive *NARS2* variants identified in 8 individuals with neonatal diabetes in our study. PM1 evidence was used to supporting level because the variants are located in the catalytic domain, which is critical for protein function but there is not sufficient evidence to establish whether the region is constrained against recessive variants. PM3 is used at supporting or moderate level as recommended by the SVI Recommendation for in trans Criterion (PM3).
