# Supplementary material for: Neonatal diabetes mellitus is a significant feature of COXPD‐24 caused by recessive NARS2 variants
Source: Diabet Med. 2025 Aug 31;42(11):e70129. doi: 10.1111/dme.70129 (PMC12535310; doi:10.1111/dme.70129)
Supplement: Supplementary file 1 — Table S1. Genes included in the targeted next‐generation sequencing panel for neonatal diabetes mellitus. Table S2. Variant classification of recessive NARS2 variants identified in 8 individuals with neonatal diabetes in our study. PM1 evidence was used to supporting level because the variants are located in the catalytic domain, which is critical for protein function but there is not sufficient evidence to establish whether the region is constrained against recessive variants. PM3 is used at supporting or moderate level as recommended by the SVI Recommendation for in trans Criterion (PM3). [file DME-42-e70129-s001.docx]

**Supplementary Table 1 |** Genes included in targeted next-generation sequencing panel for neonatal diabetes mellitus

| **Gene name** | **Transcript** |
| --- | --- |
| *ABCC8* | NM_001287174 |
| *AGPAT2* | NM_006412 |
| *BSCL2* | NM_032667 |
| *CD274* | NM_014143 |
| *COQ2* | NM_000574 |
| *COQ9* | NM_020312 |
| *EIF2AK3* | NM_004836 |
| *EIF2S3* | NM_001415 |
| *FICD* | NM_007076 |
| *FOXP3* | NM_014009 |
| *GATA4* | NM_002052 |
| *GATA6* | NM_005257 |
| *GCK* | NM_000162 |
| *GLIS3* | NM_001042413 |
| *HNF1B* | NM_000458 |
| *IER3IP1* | NM_016097 |
| *IL2RA* | NM_000417 |
| *INS* | NM_001185098 |
| *INSR* | NM_000208 |
| *KCNJ11* | NM_000525 |
| *LPL* | NM_00237 |
| *LRBA* | NM_001199282 |
| *MNX1* | NM_005515 |
| *NARS2* | NM_024678 |
| *NEUROD1* | NM_002500 |
| *NEUROG3* | NM_020999 |
| *NKX2-2* | NM_002509 |
| *ONECUT1* | NM_004498 |
| *PDIA6* | NM_005742 |
| *PDX1* | NM_000209 |
| *PTF1A* | NM_178161 |
| *RFX6* | NM_173560 |
| *SLC19A2* | NM_006996 |
| *SLC2A2* | NM_000340 |
| *STAT3* | NM_139276 |
| *TARS2* | NM_025150 |
| *WFS1* | NM_006005 |
| *YIPF5* | NM_030799 |
| *ZFP57* | NM_001109809 |
| *ZNF808* | NM_001039886 |

**Supplementary Table 2 |** Variant classification of recessive *NARS2* (NM_024678) variants identified in 8 individuals with neonatal diabetes in our study. PM1 evidence was used to supporting level because the variants are located in the catalytic domain which is critical for protein function but there is not sufficient evidence to establish whether the region is constrained against recessive variants. PM3 is used at supporting or moderate level as recommended by the SVI Recommendation for in trans Criterion (PM3).

| **Variant** | **In catalytic domain (PM1)** | **gnomADv4 frequency (PM2)** | **N of individuals identified with the variant (PM3)** | **REVEL (PP3)** | **ACMG criteria used for variant classification** | **Variant classification score** |
| --- | --- | --- | --- | --- | --- | --- |
| c.539C>A, p.(Thr180Asn) | Yes | 0 | 3 | 0.907 | PM1_supporting, PM2_moderate, PM3_moderate,  PP3_supporting,  PP4_supporting | **7 - Likely pathogenic** |
| c.648C>G, p.(Phe216Leu) | Yes | 0 | 4 | 0.682 | PM1_supporting,  PM2_moderate, PM3_modereate, PP3_supporting,  PP4_supporting | **7 – Likely pathogenic** |
| c.1318G>T, p.(Val440Leu) | Yes | 6.195e-7  (1 heterozygote) | 1 | 0.594 | PM1_supporting,  PM2_moderate, PM3_supporting,  PP3_supporting  PP4_supporting | **6 - Likely pathogenic** |
